# Recurrent camouflaged invasions and dispersal of an Asian freshwater gastropod in tropical Africa

**DOI:** 10.1186/s12862-015-0296-2

**Published:** 2015-03-07

**Authors:** Bert Van Bocxlaer, Catharina Clewing, Jean-Papy Mongindo Etimosundja, Alidor Kankonda, Oscar Wembo Ndeo, Christian Albrecht

**Affiliations:** Department of Animal Ecology and Systematics, Justus Liebig University Giessen, Heinrich-Buff-Ring 26-32 (IFZ), D-35392 Giessen, Germany; Departments of Paleobiology and Invertebrate Zoology, National Museum of Natural History, Smithsonian Institution, 10th and Constitution NW, Washington, DC 20560 USA; Research Unit Palaeontology, Department of Geology and Soil Science, Ghent University, Krijgslaan 281 (S8), 9000 Ghent, Belgium; Museum für Naturkunde, Leibniz Institute for Evolution and Biodiversity Science, Invalidenstraße 43, 10115 Berlin, Germany; Department of Hydrobiology and Aquaculture, University of Kisangani, BP 2012, Kisangani, D. R. Congo; Department of Hydrobiology, Official University of Ruwenzori, BP 560, Butembo, D. R. Congo

**Keywords:** Phylogeny, Morphology, Ancient Lakes Malawi and Tanganyika, Congo River, *Melanoides tuberculata*, Biogeography, Ecosystem change, Anthropogenic stressor, Feedback mechanisms

## Abstract

**Background:**

Non-indigenous taxa currently represent a large fraction of the species and biomass of freshwater ecosystems. The accumulation of invasive taxa in combination with other stressors in these ecosystems may alter the habitats to which native taxa are adapted, which could elicit evolutionary changes in native populations and their ecological interactions. Assessing ecological and evolutionary consequences of invasions simultaneously may therefore be the most effective approach to study taxa with complex invasion histories. Here we apply such an integrated approach to the cerithioid gastropod *Melanoides tuberculata*, a model system in invasion biology.

**Results:**

Molecular phylogenetics and ancestral range reconstructions allowed us to identify several independent Asian invasions in Lakes Malawi and Tanganyika, the Congo River, Nigeria and Cameroon. Some invasive *M. tuberculata* populations display much variation in shell morphology, and overlap in morphospace with *M. tuberculata* populations native to Africa. Experiments confirmed great ecophenotyic plasticity in some invasive populations, which, in combination with the overlap in disparity with native populations, masks invaders and their dispersal through Africa. Finally, the results of geographic modeling indicate that cryptic *M. tuberculata* invasions occurred primarily in densely populated areas.

**Conclusions:**

We reveal the continental nature of invasions of Asian *M. tuberculata* to Africa. Several of the affected ecosystems have high endemicity in Cerithioidea: Lake Tanganyika has an unparalleled diversity in freshwater cerithioids (>10 endemic genera) and the Congo Basin and Lake Malawi are home to the two largest endemic species clusters of *Melanoides* in Africa (~12 and ~8 species, respectively). Cerithioids perform ecologically important functions in the benthic ecosystems of African freshwaters, but invaders and ecosystem change pose risks to their native diversity. We draw suggestions for more effective conservation strategies from our integrated approach.

**Electronic supplementary material:**

The online version of this article (doi:10.1186/s12862-015-0296-2) contains supplementary material, which is available to authorized users.

## Background

Introductions of non-indigenous species are among the least reversible of human impacts on the world’s ecosystems [1]. They decrease global biodiversity via the extirpation of native faunas and biotic homogenization, and regularly cause considerable economic loss [2-5]. The world’s freshwater ecosystems are heavily affected by biological invasions, and non-indigenous taxa constitute a large fraction of the species, individuals, and biomass in such ecosystems. In North America and Europe at least 1,176 freshwater invaders have been recorded [1,6], but for tropical Africa knowledge is more limited. Nevertheless, African examples exist in a wide variety of taxa, including plants, invertebrates and vertebrates, and some of these cases illustrate the deep and far-reaching ecological impacts of invaders across various ecosystem levels. Perhaps the most renowned and ecologically devastating example from Afrotropical freshwaters comes from Lake Victoria, where the Nile Perch (*Lates niloticus*) was introduced [7]. This invasive piscivore drove many of the 500+ endemic cichlid fishes to extinction [8], and, aided by other stressors, including eutrophication and blooms of the invasive water hyacinth *Eichhornia*, caused rapid and wholesale ecosystem change in the lake [9]. Although the introduction of the Nile Perch can be considered economically lucrative, it increased economic stratification and because the fish is locally unaffordable [10] it perhaps did not increase availability of animal protein to the local population.

The case of Lake Victoria substantiates (1) that freshwater biodiversity regularly faces the effects of several anthropogenic stressors at once [11,12], (2) that the effects of invasions can be particularly devastating in isolated, eco-insular systems such as the African Great Lakes [13,14], and (3) that biological invasions may drastically alter ecological and evolutionary patterns in ecosystems and their native biota [15,16]. Hence, the case underscores the relevance of approaches that investigate invasion dynamics and effects simultaneously at various biological levels (e.g. invasive populations, community effects, emerging continental patterns), especially for taxa with complex invasion histories.

Here we study African populations of the cerithioid gastropod *Melanoides tuberculata* (Müller, 1774), a model system in invasive biology [17]. The species is polyploidy and has a mixed reproductive system with parthenogenetic and sexual reproduction [18]. Sexual reproduction is rare, resulting in the persistence of entire genotypes for many generations [17,18]. Because of the predominant parthenogenetic reproduction, local populations of a genetic clone usually display limited morphological variation [18]. As a result, previous authors [18-22] have characterized clonal populations as morphs, and have assigned three-letter codes to these morphs.

Indigenous strains of *M. tuberculata* occur on the African continent but around the 1980s, an Asian lineage of *M. tuberculata* (LMI morphs) invaded Lake Malawi [21]. This invasion remained long unnoticed, although the invasive morphs have a characteristic morphology, because morphological variation within and between indigenous *M. tuberculata* morphs is poorly understood, so that native lineages camouflage the invader’s presence. Consequently, variation in other indigenous African populations of *M. tuberculata* may likewise obscure the presence of invaders, which hampers our understanding of colonization history and therewith appropriate monitoring and conservation strategies. Moreover, the invader became fully established in Lake Malawi [23], and recent changes in ecological interactions have resulted in marked responses in the benthic community [24]. The camouflaged invasion and its ecological and evolutionary consequences in Lake Malawi urge further investigations of the invasion history of *M. tuberculata* on the African continent. Because *M. tuberculata* has a notorious invasion history in the Neotropics [17,20], and because knowledge on its African invasions is comparatively limited, we investigate the species with an integrated approach.

First, we produce a backbone phylogeny that allows assessing phylogeographic patterns, evolutionary history and therewith molecular screening of new *Melanoides* collections spanning the East African Rift and surroundings for potential invaders. Morphological studies of wild-collected and lab-bred specimens were conducted to obtain insights into the variability of shell morphology in *M. tuberculata*, and combined with the phylogeny into genotype-phenotype relationships. Integration of morphological and molecular data is particularly important because some gastropods have been misperceived as invasive [25], and because phenotypic plasticity and genetic variation influence dispersal capabilities and, hence, ‘invasiveness’ [15,26]. Moreover, a better understanding of shell morphology would facilitate morphological identifications of invasive strains in the field. Using geographical modeling we also investigate how humans and anthropogenic stressors affect the invasion history of *M. tuberculata* in Africa. Finally, we discuss the implications of our findings to conservation strategies for African freshwater habitats.

## Methods

### Sampling and experimental procedures

**Figure 1:**
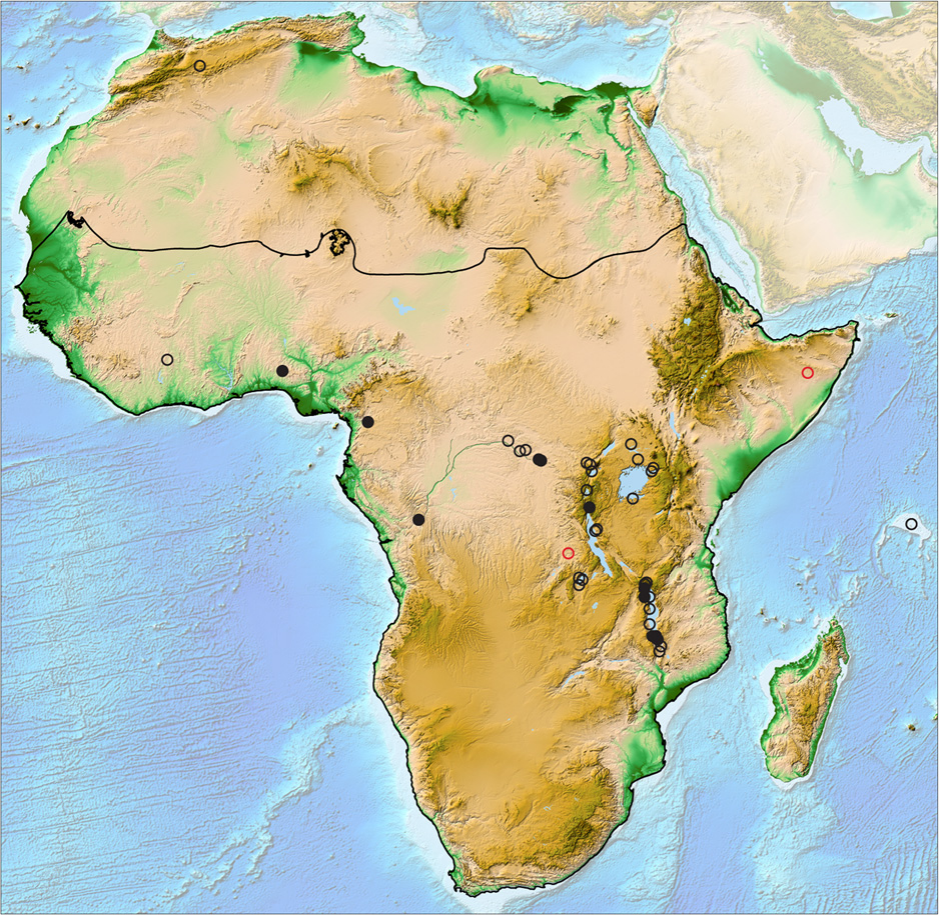
**Map of Africa with localities of** ***Melanoides*** **populations included in our study.** The genus occurs in a wide variety of water bodies throughout Africa (rivers, ponds, lakes), but is absent from substantial areas, e.g. in the Sahara it has scattered occurrences in oasis lakes. Solid symbols indicate invasive *M. tuberculata* populations, open ones native *Melanoides* populations. Two red symbols indicate approximate localities of GenBank material. A solid black line delimits sub-Saharan Africa as defined for modeling purposes following [28]. Map modified from Amante and Eakins [29].

We collected material from the Congo River, its tributaries and the Lake Edward region of the Democratic Republic of Congo (DRC), from Lake Tanganyika in Burundi, Lake Malawi and the Shire River in Malawi, Lake Kivu in Rwanda, and Lakes Kyoga and Edward, and the Victorian Nile River in Uganda (all between 2006 and 2012), and supplemented this material with previously published samples (Table 1; Figure 1). Material collected for phylogenetic studies was preserved in 80% EtOH. Specimens of one *Melanoides* population (*n* = 40; 09-032; CD05-CD06) from Kisangani (DRC) were maintained and bred in laboratory tanks after which the shell morphology of wild-caught parents, lab-bred F_1_ offspring, and other populations of *M. tuberculata* were compared. For the experiment we used a 100 l tank that had been set up before for common garden experiments with endemic gastropods from Lake Malawi (seeded with Malawi sand and water; see [27]). Water conditions reflected those of Lake Malawi (pH ~8.0; T = ~26°C), but with a higher amount of dissolved oxygen (~6.0 mL/L instead of 3.5-4.0 mL/L), and a higher electrical conductivity (increased bicarbonate hardness; ~1,800 μS/cm instead of ~260 μS/cm in the lake) to prevent shell corrosion. These values are somewhat dissimilar from those measured in waters in the Kisangani area (09-032; pH ~6.0-7.0; T = ~24°C, conductivity ~70 μS/cm).

**Table 1:** **Identification, collecting information, and NCBI GenBank accession numbers for specimens included in molecular analyses**

						**GenBank numbers**		
**Specimen code**	**Taxon**	**Country**	**Locality**	**Year**	**Prep No.**	**COI**	**16S**	**Morph**
BI01/1	*M. tuberculata*	Burundi	Lake Tanganyika	2012	19052	KP774674	KP774633	**BIT**
BI01/2	*M. tuberculata*	Burundi	Lake Tanganyika	2012	21072	KP774675	KP774634	**BIT**
BI01/3	*M. tuberculata*	Burundi	Lake Tanganyika	2012	21073	KP774676	KP774635	**BIT**
BI01/4	*M. tuberculata*	Burundi	Lake Tanganyika	2012	21074	KP774677	KP774636	**BIT**
BI01/5	*M. tuberculata*	Burundi	Lake Tanganyika	2012	21096	KP774678	KP774637	**BIT**
BI01/6	*M. tuberculata*	Burundi	Lake Tanganyika	2012	21097	KP774679	KP774638	**BIT**
BI01/7	*M. tuberculata*	Burundi	Lake Tanganyika	2012	21098	KP774680	KP774639	**BIT**
CD15/1*	*M. anomala*	DR Congo	Mwati River, Shaba Province (= Katanga)			AY958726		**n.a.**
CD15/2*	*M. anomala*	DR Congo	Kiseru River, Shaba Province (=Katanga)			AY958727		**n.a.**
CD01/1	*M. tuberculata*	DR Congo	Kisangani, Makiso (09-031)	2009	16491	KP774681	KP774640	**CDI**
CD02/1	*M.* cf. *liebrechtsi*	DR Congo	Congo-Itimbiri (Engengele; 09-023)	2009	19042	KP774682	KP774641	**n.a.**
CD02/2	*M.* cf. *liebrechtsi*	DR Congo	Congo-Itimbiri (Engengele; 09-023)	2009	19043	KP774683		**n.a.**
CD02/3	*M.* cf. *nsendweensis*	DR Congo	Congo-Itimbiri (Engengele; 09-023)	2009	19044	KP774684	KP774642	**n.a.**
CD03/1	*M.* cf. *liebrechtsi*	DR Congo	Aruwimi River (Basoko; 09-027A)	2009	19048	KP774685		**n.a.**
CD04/1	*M.* cf. *liebrechtsi*	DR Congo	Aruwimi River (Basoko; 09-027B)	2009	19046	KP774686	KP774643	**n.a.**
CD05/1	*M. tuberculata*	DR Congo	Kisangani, Makiso (09-032A)	2009	16493	KP774687		**CDI**
CD06/1	*M. tuberculata*	DR Congo	Kisangani, Makiso (09-032B)	2009	16495	KP774688	KP774644	**CDI**
CD07/1	*M. tuberculata*	DR Congo	Kisangani, Kitenge (09-033)	2009	16499	KP774689	KP774645	**CDI**
CD08/1	*M. cf. liebrechtsi*	DR Congo	Aruwimi River (Yakoyo; KM10-011)	2010	19040	KP774690	KP774646	**n.a.**
CD09/1	*M. tuberculata*	DR Congo	Ngenengene River	2012	18588	KP774691	KP774647	**CDI**
CD09/2	*M. tuberculata*	DR Congo	Ngenengene River	2012	18589	KP774692	KP774648	**CDI**
CD10/1	*M. tuberculata*	DR Congo	Avokoko River	2012	18592	KP774693	KP774649	**CDI**
CD10/2	*M. tuberculata*	DR Congo	Avokoko River	2012	18593	KP774694	KP774650	**CDI**
CD10/3	*M. tuberculata*	DR Congo	Avokoko River	2012	18594	KP774695		**CDI**
CD10/4	*M. tuberculata*	DR Congo	Avokoko River	2012	18595	KP774696	KP774651	**CDI**
CD11/1	*M. tuberculata*	DR Congo	Kpalala River	2012	18596	KP774697	KP774652	**CDI**
CD11/2	*M. tuberculata*	DR Congo	Kpalala River	2012	18597	KP774698	KP774653	**CDI**
CD11/3	*M. tuberculata*	DR Congo	Kpalala River	2012	18598	KP774699	KP774654	**CDI**
CD11/4	*M. tuberculata*	DR Congo	Kpalala River	2012	18599	KP774700	KP774655	**CDI**
CD12/1	*M. tuberculata*	DR Congo	Lake Edward	2010	18606	KP774701	KP774656	**CLE**
CD13/1	*M. tuberculata*	DR Congo	Taliha River	2010	18608	KP774702	KP774657	**CTA**
CD14/1	*M. tuberculata*	DR Congo	Semliki River	2010	18610	KP774703		**CSM**_**1**_
CD14/2	*M. tuberculata*	DR Congo	Semliki River	2010	18611	KP774704	KP774658	**CSM**_**2**_
PF01/1*	*M. tuberculata*	French Polynesia		1998		AF236072		**FPO**
PF02/1*	*M. tuberculata*	French Polynesia		1998		AF236071		**FPO**
IL01/1*	*M. tuberculata*	Israel	Ilan	1993		AY575994		**ISR**
KE01/1*	*M. tuberculata*	Kenya	Lake Victoria			AY791913	AY791931	**KLV**
MW01/1*	*M. tuberculata*	Malawi	Kambiri Point, Lake Malawi	99-02		AY575992		**LMI**_**1**_
MW02/1*	*M. tuberculata*	Malawi	Makakola, Lake Malawi	99-02		AY575980		**LMI**_**4**_
MW03/1*	*M. tuberculata*	Malawi	Cape Maclear, Lake Malawi	99-02		AY575985		**LMI**_**2**_
MW03/13	*M. tuberculata*	Malawi	Cape Maclear, Lake Malawi	2006	21086	KP774705	KP774659	**LMI**
MW03/14	*M. tuberculata*	Malawi	Cape Maclear, Lake Malawi	2006	21087	KP774706		**LMI**
MW03/15	*M. tuberculata*	Malawi	Cape Maclear, Lake Malawi	2006	21090	KP774707	KP774660	**LMN**
MW04/1*	*M. tuberculata*	Malawi	Mkungula, Lake Malombe	99-02		AY575990		**LMN**_**3**_
MW05/1*	*M. tuberculata*	Malawi	Nkhata Bay, Lake Malawi	99-02		AY575993		**LMN**_**1**_
MW05/2*	*M. tuberculata*	Malawi	Nkhata Bay, Lake Malawi	99-02		AY575998		**LMN**_**1**_
MW06/1*	*M. tuberculata*	Malawi	Southern part of Lake Malawi	2002		AY791912		**MAC**_**1**_
MW10/1	*M. tuberculata*	Malawi	Monkey Bay, Lake Malawi	2006	21066	KP774708	KP774661	**LMI**
MW10/2	*M. tuberculata*	Malawi	Monkey Bay, Lake Malawi	2006	21080	KP774709	KP774662	**LMI**
MW10/3	*M. tuberculata*	Malawi	Monkey Bay, Lake Malawi	2006	21081		KP774663	**LMN**
MW11/1	*M. tuberculata*	Malawi	Chipoka, Lake Malawi	2006	21068	KP774710	KP774664	**LMI**
MW11/2	*M. tuberculata*	Malawi	Chipoka, Lake Malawi	2006	21069	KP774711	KP774665	**LMI**
MW12/1	*M. tuberculata*	Malawi	Zalewa, Shire River	2006	21070	KP774712	KP774666	**LMI**
MW13/1	*M. tuberculata*	Malawi	Chilumba, Lake Malawi	2006	21082		KP774667	**LMI**
MW13/2	*M. tuberculata*	Malawi	Chilumba, Lake Malawi	2006	21084	KP774713		**LMN**
MW14/1	*M. tuberculata*	Malawi	Karonga, Lake Malawi	2006	21093		KP774668	**LMI**
MW03/2*	*M. polymorpha*	Malawi	Cape Maclear, Lake Malawi			AY958744		**MP**_**D**_
MW03/3*	*M. polymorpha*	Malawi	Cape Maclear, Lake Malawi			AY958729		**MP**_**C**_
MW03/4*	*M. polymorpha*	Malawi	Cape Maclear, Lake Malawi			AY958731		**MP**_**E**_
MW03/5*	*M. polymorpha*	Malawi	Cape Maclear, Lake Malawi			AY958747		**MP**_**D**_
MW03/6*	*M. polymorpha*	Malawi	Cape Maclear, Lake Malawi			AY958730		**MP**_**B**_
MW03/7*	*M. polymorpha*	Malawi	Cape Maclear, Lake Malawi			AY958728		**MP**_**C**_
MW03/8*	*M. polymorpha*	Malawi	Cape Maclear, Lake Malawi			AY958742		**MP**_**A**_
MW03/9*	*M. polymorpha*	Malawi	Cape Maclear, Lake Malawi			AY958746		**MP**_**D**_
MW03/10*	*M. polymorpha*	Malawi	Cape Maclear, Lake Malawi			AY958743		**MP**_**A**_
MW03/11*	*M. polymorpha*	Malawi	Cape Maclear, Lake Malawi			AY958745		**MP**_**D**_
MW03/12*	*M. polymorpha*	Malawi	Cape Maclear, Lake Malawi			AY958732		**MP**_**E**_
MW05/3*	*M. polymorpha*	Malawi	Nkhata Bay, Lake Malawi			AY958756		**MP**_**I**_
MW05/4*	*M. polymorpha*	Malawi	Nkhata Bay, Lake Malawi			AY958733		**MP**_**K**_
MW05/5*	*M. polymorpha*	Malawi	Nkhata Bay, Lake Malawi			AY958736		**MP**_**L**_
MW05/6*	*M. polymorpha*	Malawi	Nkhata Bay, Lake Malawi			AY958752		**MP**_**J**_
MW05/7*	*M. polymorpha*	Malawi	Nkhata Bay, Lake Malawi			AY958740		**MP**_**I**_
MW05/8*	*M. polymorpha*	Malawi	Nkhata Bay, Lake Malawi			AY958738		**MP**_**I**_
MW05/9*	*M. polymorpha*	Malawi	Nkhata Bay, Lake Malawi			AY958734		**MP**_**G**_
MW05/10*	*M. polymorpha*	Malawi	Nkhata Bay, Lake Malawi			AY958741		**MP**_**J**_
MW05/11*	*M. polymorpha*	Malawi	Nkhata Bay, Lake Malawi			AY958739		**MP**_**I**_
MW05/12*	*M. polymorpha*	Malawi	Nkhata Bay, Lake Malawi			AY958737		**MP**_**H**_
MW05/13*	*M. polymorpha*	Malawi	Nkhata Bay, Lake Malawi			AY958735		**MP**_**L**_
PH01/1*	*M. tuberculata*	Philippines	Luzon; mountain stream N. of Iba	2000		AY456564		**PHI**_**1**_
RW01/1	*M. tuberculata*	Rwanda	Lake Kivu	2010	19037	KP774714	KP774669	**RLK**
RW01/2	*M. tuberculata*	Rwanda	Lake Kivu	2010	19039	KP774715		**RLK**
SG01/1*	*M. tuberculata*	Singapore	Lower Selatar Reservoir	2003		AY575978		**LSR**
SG02/1*	*M. tuberculata*	Singapore	Napier Road	2003		AY575972		**NAP**
SG03/1*	*M. tuberculata*	Singapore	Pandan Reservoir	2003		AY575974		**PAN**
SG03/2*	*M. tuberculata*	Singapore	Pandan Reservoir	2003		AY575975		**PAN**
SG04/1*	*M. tuberculata*	Singapore	Upper Selatar Reservoir	2003		AY575977		**USR**
SG04/2*	*M. tuberculata*	Singapore	Upper Selatar Reservoir	2003		AY575979		**USR**
SG05/1*	*Thiara scabra*	Singapore	Lower Selatar Reservoir	2003		AY958758		n.a.
SG05/2*	*Tarebia granifera*	Singapore	Lower Selatar Reservoir	2003		AY958760		**n.a.**
SG05/3*	*Tarebia granifera*	Singapore	Chinese Garden	2003		AY958761		**n.a.**
SG05/4*	*Tarebia granifera*	Singapore	Lower Selatar Reservoir	2003		AY958762		**n.a.**
SG05/5*	*Tarebia granifera*	Singapore	Napier Road	2003		AY958763		**n.a.**
SG05/6*	*Tarebia granifera*	USA	Miami, Florida	2003		AY958764		**n.a.**
SO01/1*	*M. tuberculata*	Somalia	Eil Spring	1992		AY575973		**SOM**
TZ01/1*	*M. tuberculata*	Tanzania	Mwanza Gulf, Lake Victoria	2002		AY575996		**VIC**
TZ01/2*	*M. tuberculata*	Tanzania	Mwanza Gulf, Lake Victoria	2002		AY575995		**VIC**
TZ02/1*	*M. tuberculata*	Tanzania	Itungi, Lake Malawi	2002		AY791909	AY791910	**TAI**
TZ03/1*	*M. admirabilis*	Tanzania	Malagarasi River			AY958725		**n.a.**
UG01/1	*M. tuberculata*	Uganda	Jinja, Victoria Nile	2010	18600	KP774716	KP774670	**UNI**
UG02/1	*M. tuberculata*	Uganda	Lake Kyoga	2010	18602	KP774717	KP774671	**ULK**
UG03/1	*M. tuberculata*	Uganda	Lake Edward	2010	18604	KP774718	KP774672	**ULE**
ZM01/1*	*M. imitatrix*	Zambia	Isokwe Island, Lake Mweru			DQ995480		**n.a.**
ZM02/1*	*M. imitatrix*	Zambia	Isokwe Island, Lake Mweru			DQ995479		**n.a.**
ZM03/1*	*M. mweruensis*	Zambia	Kilwa Island, Lake Mweru			DQ995481		**n.a.**
TZ06/1*	*M. admirabilis*	Tanzania	Malagarasi River	2000		AY456561		**n.a.**
MW05/14*	*M. woodwardi*	Malawi	Nkhata Bay, Lake Malawi	2002			AY791903	WOO
MW07/1*	*M. turritispira*	Malawi	Nkhota Kota, Lake Malawi	2002			AY791908	TUR
CA01/1*	*M. tuberculata*	Cameroon	Eseka				AY791914	**CAE**
MW08/1*	*M. tuberculata*	Malawi	Chembe, Lake Malawi	2002			AY791915	**MAC**_**1**_
TZ04/1*	*M. truncatelliformis*	Tanzania	Mwaya, Lake Malawi	2002			AY791917	TRU
TZ04/2*	*M. simonsi*	Tanzania	Mwaya, Lake Malawi	2002			AY791918	**TAM**
TZ05/1*	*M. pupiformis*	Tanzania	Kiwira, Lake Malawi	2002			AY791920	PUB
TZ04/3*	*M. polymorpha*	Tanzania	Mwaya, Lake Malawi	2002			AY791924	POL
TZ05/2*	*M. nodicincta*	Tanzania	Kiwira, Lake Malawi	2002			AY791927	**TAK**
TZ04/4*	*M. magnifica*	Tanzania	Mwaya, Lake Malawi	2002			AY791928	MAG
TZ05/3*	*M. nyassana*	Tanzania	Kiwira, Lake Malawi	2002			AY791933	NYA
CD15/1*	*M. tuberculata*	DR Congo	Kinshasa	1994			AY283067	**ZAK**
FW01/1*	*M. amabilis*	French West Indies	Martinique	2000			AY283068	**n.a.**
FW01/2*	*M. tuberculata*	French West Indies	Martinique	1999			AY283071	PDC
IC01/1*	*M. tuberculata*	Ivory Coast	Bouaké	1995			AY283072	BOU
OM01/1*	*M. tuberculata*	Oman	Afilayia	2000			AY283073	OMW
MO01/1*	*M. tuberculata*	Morocco	Figuig	1993			AY283074	MOF
FW01/3*	*M. tuberculata*	French West Indies	Martinique	1999			AY283075	FAL
KE02/1*	*M. tuberculata*	Kenya	Kisumu, Lake Victoria	2000			AY283076	KIS
SY01/1*	*M. tuberculata*	Seychelles		2000			AY283077	ND
CO01/1*	*M. tuberculata*	Colombia	San Jeronimo	1999			AY283078	COL
US01/1*	*M. tuberculata*	USA	Florida	2000			AY283079	**BCI**
PE01/1*	*M. tuberculata*	Peru	Tumbes	1994			AY283080	**TUM**
IN01/1*	*M. tuberculata*	Indonesia	Lombok	2000			AY283081	**ND**
PF03/1*	*M. tuberculata*	French Polynesia	Moorea	1999			AY283083	MOO
VE01/1*	*M. tuberculata*	Venezuela	Choroni	2001			AY283084	CHO
US02/1*	*M. tuberculata*	USA	Florida	2000			AY010517	**USF**
AR01/1*	*M. tuberculata*	Argentina	Misiones				EF523385	**ARG**
MW09/1*	*M. tuberculata*	Malawi	Lake Malawi	99-00			AY456616	**LMN**
US03/1*	*M. tuberculata*	USA					AF101006	USX
PH01/2*	*M. tuberculata*	Philippines	Luzon; mountain stream N. of Iba	2000			AY456618	**PHI**_**2**_
TZ06/2*	*M. admirabilis*	Tanzania	Malagarasi River	2000			AY456615	n.a.
MU01/1	*Thiara scabra*	Mauritius	Pamplemousses, Citron River	2013	20294	KP774719	KP774673	**n.a.**
PF04/1*	*Tarebia granifera*	French Polynesia	Tahiti	1998			AY283069	n.a.
ZA01/1*	*Lavigeria grandis*	Zambia	Lake Tanganyika, Kumbula Island	1999		AY456539		**n.a.**
ZA01/1*	*Lavigeria grandis*	Zambia	Lake Tanganyika, Kasenga Point	1999			AY456594	**n.a.**
SP01/1*	*Hydrobia glyca*	Spain	San Vicente de la Barquera			AF467653		**n.a.**
SP01/2*	*Hydrobia glyca*	Spain	San Fernando				AF478397	**n.a.**

When species determination was ambiguous (e.g. for juveniles), we added cf. to the species names. Shells were usually broken during DNA isolation; tissue and shells or fragments are stored at the Justus Liebig University, Giessen and indicated with DNA preparation numbers (UGSB collection). *indicates material for which sequences were obtained from NCBI GenBank. Material that was included in the final combined dataset is highlighted with bold morph codes (last column).

### Morphological characterization

*Melanoides* specimens were identified based on shell morphology using the relevant literature [30,31]. Shell morphology was studied directly and after cleaning with bleach. Morphological comparisons were performed independently by B.V.B. and C.C. using a modified version (1 extra trait, some additional trait values) of the categorical shell-morphological character scoring system of Facon et al. [20]. Modifications allow coding the sampled endemic *Melanoides* species of the DRC for comparison. As mentioned, the predominantly parthenogenetic reproduction of *M. tuberculata* usually results in limited intrapopulation variation in shell morphology [18]. Discrete morphs can hence be recognized and the morphological scoring system is considered to be generally robust for delimiting parthenogenetic lineages of *M. tuberculata*, although closely related morphs occasionally display strikingly different shell features [20,21]. Characters with multiple states were converted to arithmetic means and morphological scores were converted into a distance matrix that was ordinated with non-metric multidimensional scaling (nmMDS) in three dimensions following the guidelines of Van Bocxlaer and Schultheiß [32]. We used the packages MASS version 7.3-27 [33] and vegan version 2.0-10 [34] in R version 3.0.1 [35] for nmMDS.

### Phylogenetic analyses

Genomic DNA was isolated using the CTAB protocol [36]. Two gene markers were used: (1) mitochondrial cytochrome *c* oxidase subunit I (COI), and the (2) mitochondrial large ribosomal subunit (mtLSU rRNA or 16S). DNA, specimen and image vouchers of newly sequenced material (49 specimens) were deposited at the Justus Liebig University’s Systematics and Biodiversity collection (UGSB); data on other specimens (59 COI and 37 16S sequences) was retrieved from NCBI GenBank (Table 1).

COI sequences were obtained utilizing the forward primer LCO1490 [37], and the reverse primers HCO2198 [37] or COX-B7R [38]. For amplification of partial 16S DNA we used the forward and reverse primers (16Sar-L and 16Sbr-H) from Palumbi et al. [39]. PCR cycling conditions are specified in Additional file 1: Table S1. Bidirectional DNA sequencing was performed on a 16-capillary 3730*xl* Genetic Analyzer (Applied Biosystems). New sequences were deposited in GenBank (Table 1). The first basepairs (bp) behind the 3’ end of each primer were difficult to read. We therefore trimmed each sequence, leaving a 580 bp-long overlapping COI fragment and a 451-455 bp-long 16S fragment. The protein-coding COI sequences were unambiguously aligned using ClustalW implemented in BioEdit 7.0.8.0 [40]. We aligned 16S fragments using the multiple sequence alignment program PRANK [41] with empirical base frequencies, the Tamura-Nei substitution model and default settings for gap penalties. Ambiguously aligned sites (posterior probability of the obtained structure state ≤0.8) were excluded from subsequent analyses. Inclusion of unknown states for shorter 16S fragments obtained from GenBank caused alignment problems and the deletion of 70 bp, many of which were informative. Therefore, we decided to drop these shorter-fragment sequences from our dataset (*n* = 21; Table 1), resulting in a 452 bp long 16S alignment for 63 haplotypes.

No substantial substitutional saturation was found in the COI and 16S datasets upon testing with DAMBE version 5.2.73 [42]. The final dataset (COI + 16S) contained a total of 79 unique sequences (including one of *Thiara scabra*, four of *Tarebia granifera*, and the outgroup taxa *Hydrobia glyca* and *Lavigeria grandis*; see Table 1). Substitution models were examined for individual partitions with jModelTest 0.1.1 [43] using corrected Akaike and Bayesian Information Criteria (AICc and BIC, respectively). The best supported substitution model for the COI dataset was HKY + I + G (AICc and BIC) and for the 16S alignment GTR + G (AICc) or HKY (+ I) + G (BIC; same support with or without invariant sites). The concatenated dataset was analyzed in BEAST version 1.7.4 [44] using a Yule speciation process, a total of 40,000,000 generations (with sampling each 1,000th generation), and a burn-in of 20,001. Independent phylogenetic analyses were performed using strict and uncorrelated lognormal relaxed clock models (with 1.0 as relative rate). Effective sample size (ESS) values of all major parameters within the BEAST analyses were visualized and checked in Tracer version 1.5.0 [44]. These checks demonstrated that the ESS values for ‘prior’ and ‘posterior’ were unacceptably low when the GTR + G model was used for the 16S partition, which may indicate that the model was overparameterized. Hence, we decided to use the still well-supported but less complex HKY + I + G model for each partition in the concatenated dataset which produced good ESS values (>200) for all parameters. Bayes Factor (BF) analysis was performed in Tracer and likelihood values based on 1,000 bootstrap replicates were evaluated to select the appropriate clock model (following criteria of Kass and Raftery [45] and Suchard et al. [46]). To examine potential conflicts among the COI and 16S partitions each dataset was also analyzed individually in BEAST following the conditions described above (e.g. HKY + I + G model).

To examine invasions and to get a better insight into the geographic distribution of the ingroup taxa we performed ancestral range reconstructions with the concatenated COI + 16S dataset. For transparency and to avoid circularity we only included actual occurrences of the terminal taxa in the dataset, regardless of whether the material is known to be invasive in the area of occurrence. For example, specimens from the Neotropics are coded as American, even though the specimens represent well-documented Asian invasions [17,20]. Analyses were performed in R using the packages ape 3.1-4 [47] and geiger 2.0.3 [48], and independently with Lagrange version 20130526 [49]. For the first, regions were coded and states were reconstructed using maximum likelihood estimation and marginal reconstruction. For Lagrange a presence-absence matrix was created and combined with the phylogeny in the Lagrange configurator (http://www.reelab.net/lagrange/configurator/index) to create the input file, which was analyzed using default settings.

### Geographical modeling

Anthropogenic influences on the colonization history of invasive *M. tuberculata* in Africa were assessed with spatial modeling. We used human population size as a proxy of the variety of anthropogenic effects, e.g. human-mediated dispersal and anthropogenic changes in habitats (pollution, use of natural resources, and climate change), that humans have on natural environments in a particular region. Specifically, we investigated the null hypothesis that localities with recorded invasions represent a random sample with respect to human population. We used NASA SEDAC’s gridded population of the World data (GPWv3; count data, estimates for 2010) [50] with a spatial resolution of ~21 km^2^, and extracted data for sub-Saharan Africa (as defined in [28]) as an estimator of anthropogenic stressors. We compared human population counts for cells where invasive *Melanoides* specimens are recorded to the counts for all grid cells (*n* = 988,224) and assessed the probability of observed patterns in two ways. First, we divided all grid cells in two groups; the first group contains all cells where human population counts are smaller than the minimum count in grid cells with invasive gastropods; the second group has all counts higher than this minimum. We then calculated the statistical chance for the observed number of invasions to occur in the grid cells where they were observed assuming human population density is not affecting invasions. Second, we drew 10,000 random samples of grid cells from sub-Saharan Africa, each with a size equal to the number of grid cells with observed invasions and summed the human population counts of these grid cells. With these random samples we built a frequency distribution against which we plotted the summed human population counts from grid cells with invasions. Spatial modeling was performed in R 3.0.1 using the packages raster 2.2-31 [51], rgdal 0.9-1 [52], dismo 0.9-3 [53] and MASS.

### Systematics

The nominal, endemic *Melanoides* species of Lake Malawi have been aggregated by some authors [22,54] into a ‘*M. polymorpha* complex’, even though distinct morphs can be discerned in the modern [31,55] and fossil faunas of Lake Malawi [56]. We have not altered previous assignments of specimens in GenBank, but consider the traditional species valid until a formal taxonomic revision demonstrates otherwise.

## Results

### Phylogenetic analysis

**Figure 2:**
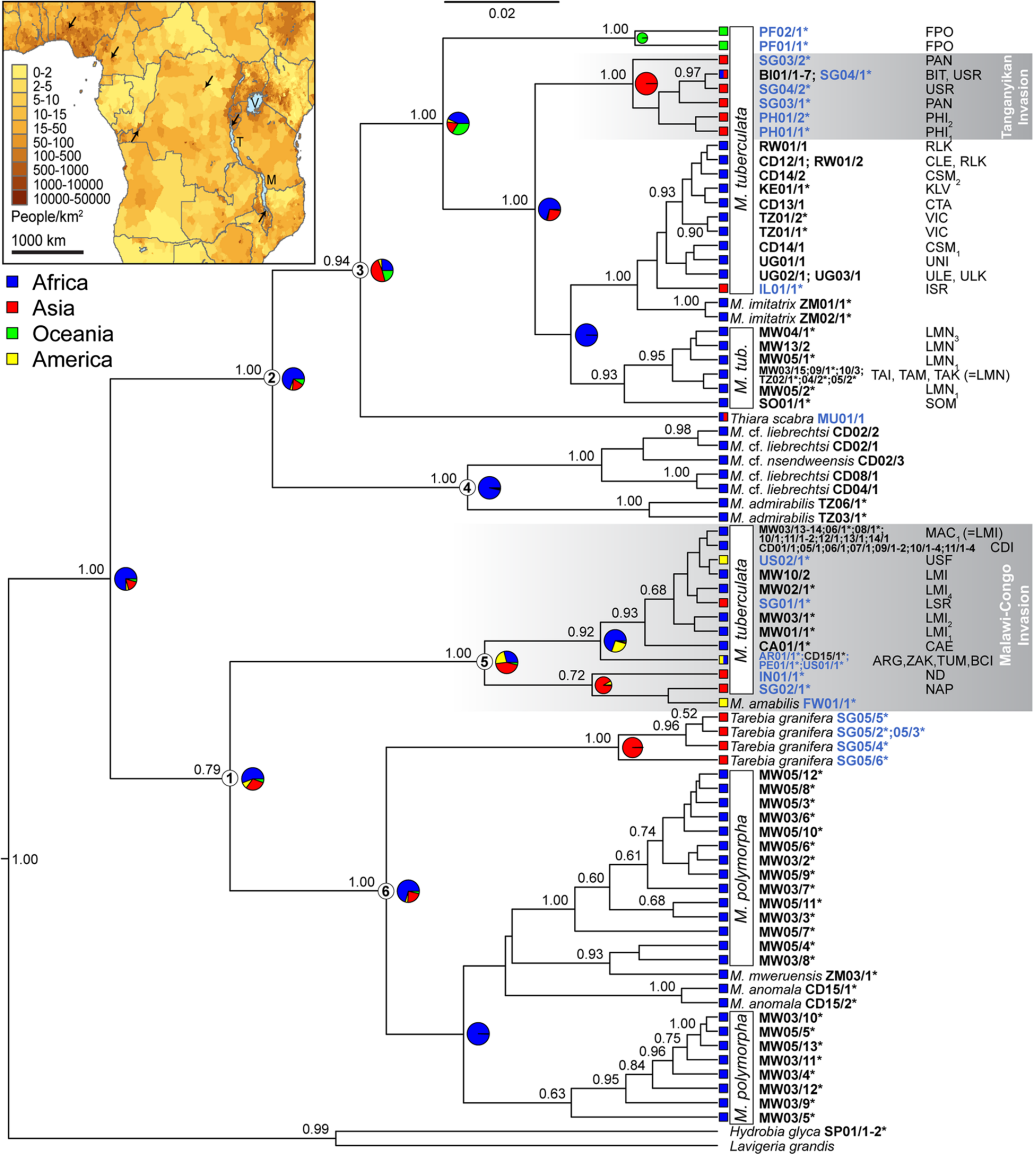
**Maximum credibility phylogeny of** ***Melanoides*** **based on mitochondrial COI and 16S data.** Bayesian posterior probabilities are given above the nodes (values <0.50 not indicated). Color coding indicates geographical range, with representation of the maximum likelihood reconstructions of ancestral geographic origin at selected nodes; black codes for terminal taxa indicate an African origin; blue ones originate from other continents. Three-letter morph codes are indicated for *Melanoides tuberculata*. The scale bar represents substitutions per site according to the applied model of sequence evolution. Clades specifically discussed in the text are labeled at their basal nodes with encircled numbers (which also facilitate comparison to Figure 3). Gradient rectangles indicate the invasions here discussed. *indicates sequences obtained from GenBank. The inset map of tropical Africa indicates that invasions (arrows) occurred in areas with high human population density; Lakes Malawi, Tanganyika and Victoria are labelled. Population data were obtained from NASA’s Socioeconomic Data and Applications Center (http://sedac.ciesin.columbia.edu; copyright ownership by the Center for International Earth Science Information Network [CIESIN]; accessed 28 March 2014).

**Figure 3:**
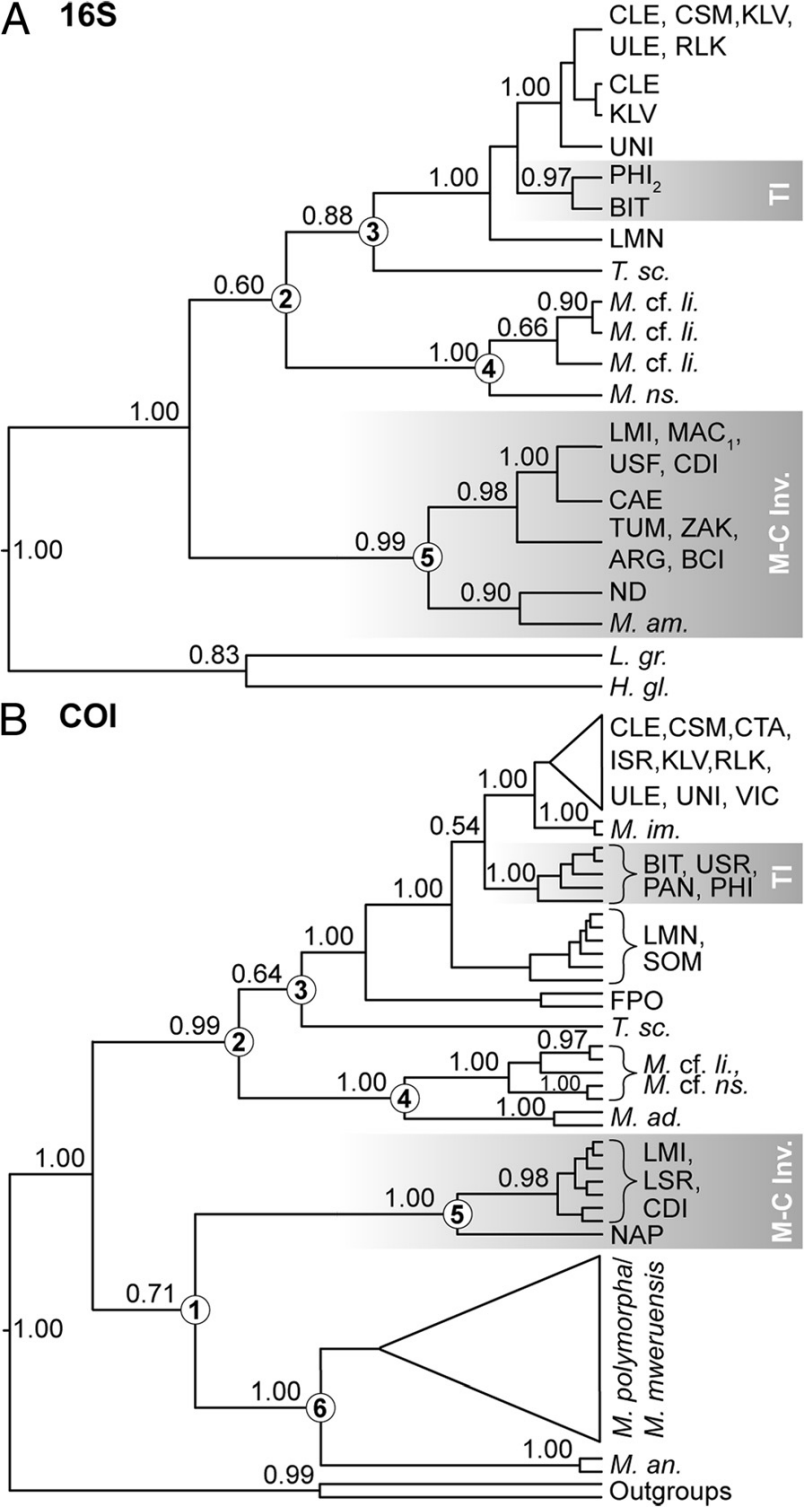
**Maximum credibility phylogenies of** ***Melanoides*** **based on mitochondrial (A) 16S and (B) COI data.** Bayesian posterior probabilities are given above the nodes (values below 0.50 not indicated). Three-letter morph codes are indicated for *Melanoides tuberculata*; other species names abbreviated. Gradient rectangles indicate the invasions here discussed (TI, M-C Inv. = Tanganyika and Malawi-Congo invasion, respectively). Both gene trees have branching patterns that are highly similar to those inferred from the concatenated dataset (Figure 2), and all trees corroborate invasions of Asian *Melanoides tuberculata* to Africa.

Phylogenetic analyses of the concatenated COI + 16S dataset (Figure 2) resulted in overall highly similar topologies to those in single fragment phylogenies (Figure 3), despite substantial differences in the sampling for both genes (Table 1). Analyses of BF revealed that the fit of uncorrelated lognormal relaxed clock models to our datasets was consistently better than that of strict clock approaches, and hence uniform rates of sequence evolution along the phylogeny were rejected. Ancestral range reconstructions in R and using Lagrange resulted in almost identical results; the first method was used for visualization; results of the second method are displayed in Additional file 2: Table S2.

Phylogenetic inference revealed a split between two clades of *Melanoides* species (Figures 2 and 3), both of which have occurrences on Africa and Asia. One of these clades (clade 1) contains predominantly endemic taxa from Lakes Malawi and Mweru and the Congo Basin, a sub-clade of Asian *M. tuberculata*, and *Tarebia granifera*; the other clade (clade 2) contains taxa endemic to the Congo Basin, other morphs of *M. tuberculata* and *Thiara scabra* (Figure 2). Our phylogeny hence indicates that *Melanoides* is paraphyletic, as *Thiara scabra* and *Tarebia granifera* cluster within *Melanoides* clades (Figure 2). Moreover, *Melanoides tuberculata*, and interestingly also the African endemic *Melanoides* species were found to be polyphyletic.

Phylogenetic reconstruction (Figure 2) reveals the occurrence of an invasive Asian morph of *M. tuberculata* in the Kisangani area (DRC) that is closely related to the LMI morphs from Lake Malawi (with a basal Bayesian Posterior Probability [BPP] support of 1.00). The invasive nature of this clade is well-supported by the ancestral range reconstruction of clade 5, certainly upon considering external evidence that American taxa within this clade represent historical Asian invasions. For consistency with the literature [17,20-22] we propose CDI as the three-letter code for this morph. Whereas some molecular variability exists in COI sequences of the LMI morphs from Lake Malawi, specimens from 5 populations of morph CDI have identical COI sequences. This CDI morph was found in 4 small rivers and swamps that discharge directly or indirectly to the Congo River and that encompass an area of 150 km^2^ (Additional file 3: Table S3). Another independent Asian invader has been sampled from Lake Tanganyika near Bujumbura (again with BPP = 1.00), and belongs to clade 2 (morph BIT; Figure 2). The invasive nature of this morph is again well-supported by ancestral range reconstructions. No Asian invasions were discovered thus far upon screening samples from the Nile drainage (Table 1). Ancestral range reconstructions for the entire ingroup suggested that an African origin is most likely.

Our phylogeny also revealed a close and strongly supported (BPP of 1.00) relationship between endemic *Melanoides* species from the Lower Congo River (downstream of Kisangani) and *M. admirabilis* from the Malagarasi River at the base of clade 2 (Figure 2).

### Experimental and morphological data

**Figure 4:**
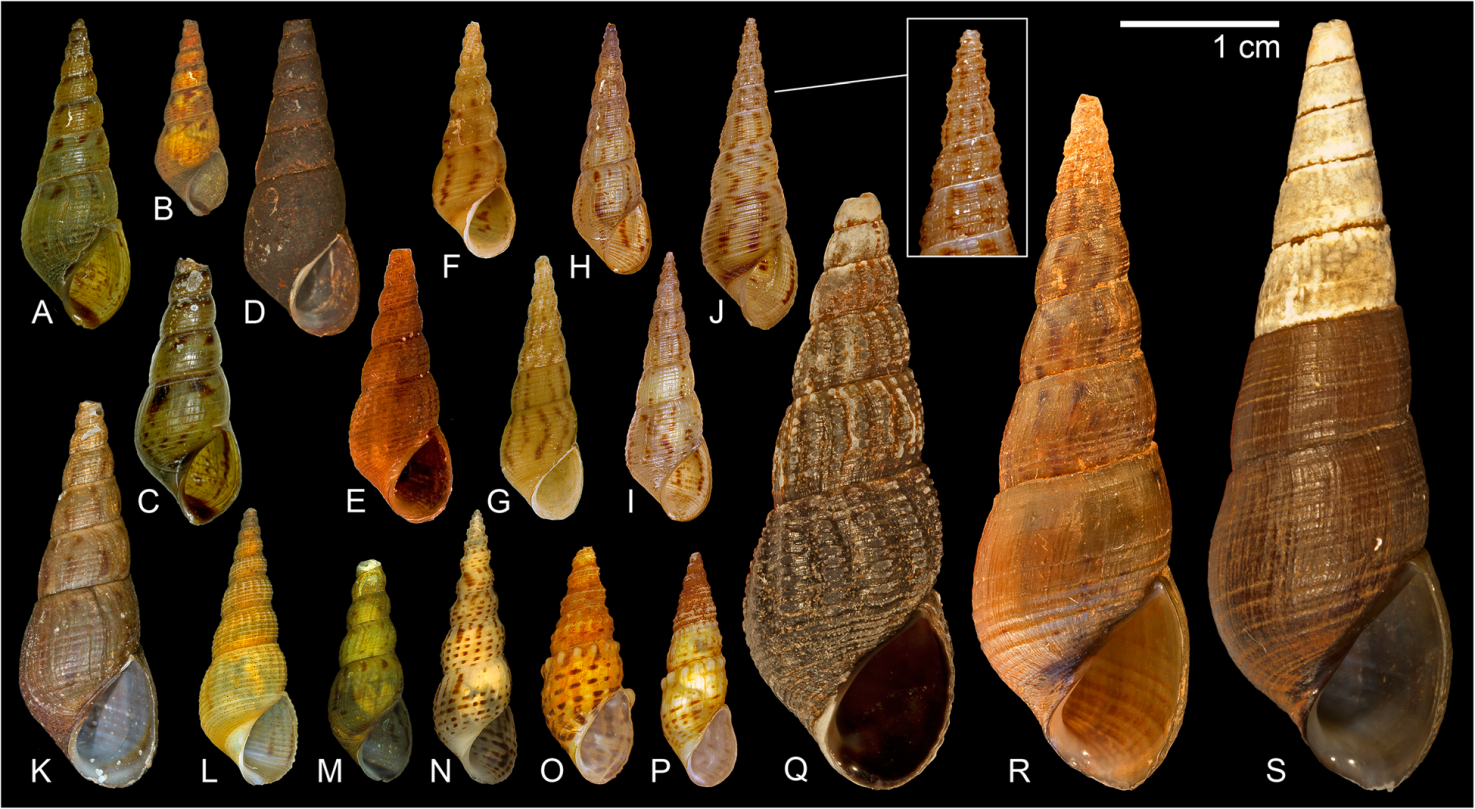
**Morphology of** ***Melanoides*** **morphs: A-C)** invasive *M. tuberculata* morph BIT from Lake Tanganyika (BI01); **D-E)** wild type of *M. tuberculata* morph CDI from the Congo River at Kisangani with organic coating (**D**: DRC09-033; **E**: DRC09-032); **F)** Lab-bred *F1* CDI specimen from population CD06 (4 months old); **G-I)** invasive *M. tuberculata* morph LMI from Chipoka at Lake Malawi (**G**: BVB-ML-08; **H-I**: UGSB 0138 [note that H-I lack a columellar band and have axial sculpture]); **J)** morph LMI from the Shire River with columellar band and axial sculpture (UGSB 1246); **K-M)** bleach-cleaned CDI specimens (**K**: CD05; **L**: CD10; **M**: CD07); **N)** native *M. tuberculata* from Lake Edward (CD12); **O-P)** to the Congo Basin endemic *M.* cf. *liebrechtsi* (**O**: CD02; **P**: CD08); **Q)** native *M. tuberculata* morph LMN from Monkey Bay at Lake Malawi (UGSB 0183); **R-S)** non-parasitized giants of the morph LMI from the same locality as **Q** (UGSB 0183).

Freshly collected *Melanoides* specimens from the Kisangani area have a very conspicuous dark-brown to black organic coating that masks most shell features. Upon bleaching, wild-caught individuals of morph CDI displayed more variability in shell features than did the LMI specimens from Lake Malawi as reported by Genner et al. [21], but lab-bred *F1* specimens of morph CDI (*n* = 10) did not and resemble these LMI morphs strikingly (Figure 4, Table 2). A columellar band is usually present in LMI morphs [21] but it is often absent in specimens of morph CDI, and whereas axial ribs were reported to be absent in LMI, they are regularly present on juvenile whorls in CDI, albeit shallow (Table 2). All wild-caught individuals from population 09-032 that we examined lacked a columellar band and some had axial ribs (Table 2), but the band was faintly present on lab-bred *F1* offspring of the population, which lacked axial sculpture, even on juvenile whorls (Figure 4F).

**Table 2:** **Categorical description of** ***Melanoides*** **species/morphs based on shell morphology using an expanded version of the scoring system of Facon et al.** [20]

		**Background colour**			**Colour patterns**				**Columellar band**		**General shape**		**Sculpture**			
**Species**	**Population code**	**IN**	**TI**	**HE**	**DO**	**SP**	**SO**	**HO**	**SH**	**SC**	**CO**	**RO**	**GR**	**CD**	**RI**	**RD**
**Clade 1**																
*M. tuberculata*	CD01/1^1^	4	5	0	0	na	na	na	1	na	1	2	2	0	3	1
*M. tuberculata*	CD01-A	2	2	0	2	2	3	1	2	2	1	2	2	0	1	1
*M. tuberculata*	CD01-B	2	4	0	2	1	2	1	1	na	1	2	2	0	3	1
*M. tuberculata*	CD05-A	1	4	0	1	1	2	1	1	na	1	2	2	0	3	1
*M. tuberculata*	CD05-B	2	1	0	2	1	2	1	1	na	1	2	1	0	0	na
*M. tuberculata*	CD06-A	1	4	0	1	2	1, 2	1	1	na	1	2	2	0	3	1
*M. tuberculata*	CD06-B	2	1	0	1	2	1, 2	1	1	na	1	2	1	0	0	na
*M. tuberculata*	CD05-06 F1	2, 3	1	0	2	2	2	1	2	2	1	2	2	0	0	na
*M. tuberculata*	CD07-A	2	2	0	1	2	2	2	2	1	1	3	1	0	3	1
*M. tuberculata*	CD07-B	2, 3	1	0	1	1	2	1	1	na	1	3	1	0	0	na
*M. tuberculata*	CD09-A	3	1	0	1	2	1	1	1	na	1	2	1	0	3	1
*M. tuberculata*	CD09-B	2	1	0	1	2	2	1	2	2	1	2	1	0	1	1
*M. tuberculata*	CD10/1-4^1^	4	5	0	0	na	na	na	1	na	1	2	3	0	3	2, 1
*M. tuberculata*	CD10-A	2	1	0	0	na	na	na	2	2	1	3	2	0	3	2
*M. tuberculata*	CD10-B	2	4	0	1	2	1	1	1	na	1	3	2	0	3	2
*M. tuberculata*	CD11/1-4^1^	4	5	0	0	na	na	na	1	na	1	2	2	0	0	na
*M. tuberculata*	LMI_1_^2^	2	1	0	2	2	2	1	2	2	1	2	2	0	0	na
*M. tuberculata*	LMI_2_^2^	2	2	0	2	2	2	1	2	2	1	2	2	0	0	na
*M. tuberculata*	LMI_3_^2^	3	2	0	2	2	3	1	3	3	1	3	2	0	0	na
*M. tuberculata*	LMI_4_^2^	2	1	0	2	2	3	1	3	3	1	2	2	0	0	na
*M. tuberculata*	MW11/1	3	2	0	2	2	2	1	1	na	1	2	2	0	1	1
*M. tuberculata*	MW12/1	3	2	0	2	2	2	1	2	2	1	2	2	0	1	1
**Clade 2**																
*M.* cf. *liebrechtsi*	CD02/1	3	1	0	2	2	2	2	1	na	2	2	2	2	2	1
*M.* cf. *liebrechtsi*	CD02/2	3	1	0	2	2	2	2	1	na	2	2	2	2	2	2
*M.* cf. *nsendweensis*	CD02/3	2	1	0	0	na	na	na	1	na	3	1	2	0	2	2
*M.* cf. *liebrechtsi*	CD03/1	2	1	0	1	2	2	2	1	na	2	2	2, 3	0	1	1
*M.* cf. *liebrechtsi*	CD03-A	2	1	0	2	2	1	2	1	na	2	2	2, 3	2	2	2
*M.* cf. *liebrechtsi*	CD03-B	3	1	0	2	2	2	2	1	na	2	1, 2	2	2	2	1
*M.* cf. *liebrechtsi*	CD04/1	2	1	0	2	2	2	2	1	na	2	2	2	2	2	2
*M.* cf. *liebrechtsi*	CD04-A	2	1	0	1	2	1	2	1	na	2	2	2	2	2	2
*M.* cf. *liebrechtsi*	CD04-B	2	1	0	2	2	2	2, 3	1	na	2	2	2	2	2	2
*M.* cf. *liebrechtsi*	CD08-A	1	4	0	1	3	2	2	1	na	1	2	1	3	2	2
*M.* cf. *liebrechtsi*	CD08-B	1	4	0	1	2	2	2	1	na	1	2	1, 2	3	2	1, 2
*M. tuberculata*	BI01/1	2, 3	1	1	1	1	2	3	2	2	1	2	1	0	3	1
*M. tuberculata*	BI01/2	2	2	1	1	1	2	3	2	3	1	2	1	0	3	1
*M. tuberculata*	BI01/3	3	2	1	1	1	2	3	2	3	1	2	1	0	3	1
*M. tuberculata*	BI01/4	2	2	1	1	1	2	3	2	2	1	2	1	0	3	1
*M. tuberculata*	MW10/3	4	5	0	0	na	na	na	1	na	3	3	3	0	2	3
*M. tuberculata*	MW13/2	3	1	0	2	2	1	2	1	na	2	3	3	0	3	3
*M. tuberculata*	MW13-A	2	1	0	0	na	na	na	1	na	2	2	3	0	3	3
*M. tuberculata*	RW01/1	1	1	0	0	na	na	na	1	na	2	3	3	0	3	3
*M. tuberculata*	RW01/2	2	1	1	2	2	2	3	1	na	2	2	2	0	1	1
*M. tuberculata*	UG01/1	2, 3	1	0	2	2	2	1	3	2	1	3	3	0	2	3
*M. tuberculata*	UG02/1	3	1	0	1	2	1	1	1	na	1	2	2	0	3	1
*M. tuberculata*	UG03/1	3	1	1	2	2	1	2	2	2	1	2, 3	2	0	1	1
*M. tuberculata*	CD12/1	3	1	0	2	1	3	1	1	na	1	3	2	0	3	1
*M. tuberculata*	CD13/1	3	1	1	1	2	2	2	1	na	1	2	1	0	3	1
*M. tuberculata*	CD14/1	1	1	0	1	2	2	2	1	na	2	2	1	0	3	1
*M. tuberculata*	CD14/2	2	1	0	2	2	2	2	2	1	1	3	2	0	3	2

Specimens were selected to cover the variation observed in populations. If the actual trait is on the border between two values (coded differently by the independent examiners), both values are indicated. Material indicated with a ^1^in the population code column was not bleached and reveals the actual appearance in the field; ^2^reflects assessments by Genner et al. [21].

Character explanation:

IN = intensity of the shell background color: (1) very pale, (2) pale, (3) medium, (4) dark.

TI = background tint of the shell: (1) yellow to brown, (2) greenish, (3) orange to reddish, (4) white, (5) brown to black.

HE = heterogeneity of the background color among different parts of a shell whorl: (0) homogeneous, (1) a distinctly darker band below the suture.

DO = overall density of reddish-brown color patterning on the whole shell, except the zone just below the sutures: (0) no patterning, (1) medium, (2) dense.

SP = type of patterning, expressed as the proportion of spots vs. flames: (0) only flames, (1) more flames than spots, (2) more spots than flames, (3) only spots.

SO = size of the individual spots/flames: (1) small spots/narrow flames, (2) medium, (3) large spots/wide flames.

HO = heterogeneity of color patterning among different parts of the whorl: (1) homogeneous, (2) slightly different patterns just below the sutures (i.e. in the subsutural zone), (3) strongly different patterns in the subsutural zone compared to those on the rest of the shell.

SH = presence and sharpness of a dark band on the axial edge of the aperture (columellar band) and along the base of the body whorl: (1) absent, (2) diffuse, (3) sharp.

SC = size of the dark band, when present: (1) narrow, (2) medium, (3) wide.

CO = conicity of the shell: (1) acute, (2) medium, (3) blunted cone.

RO = roundness of the body whorl: (1) flat, (2) slightly rounded, (3) well-rounded.

GR = spiral cords/grooves: (0) absent, (1) shallow grooves/weakly pronounced cords, (2) intermediate, (3) very deep grooves/strongly pronounced cords.

CD = subsutural spiral cord: (0) similar to the other spiral cords, (1) pronounced/swollen, but smooth, (2) pronounced/swollen with nods, (3) similar to other spiral cords but with large nods.

RI = density and width of axial ribs: (0) none, (1) a few narrow ribs, (2) a few large ribs, (3) many narrow ribs.

RD = depth of axial ribs when present: (1) shallow, (2) medium, (3) deep.

‘na’ indicates when the trait is not applicable.

**Figure 5:**
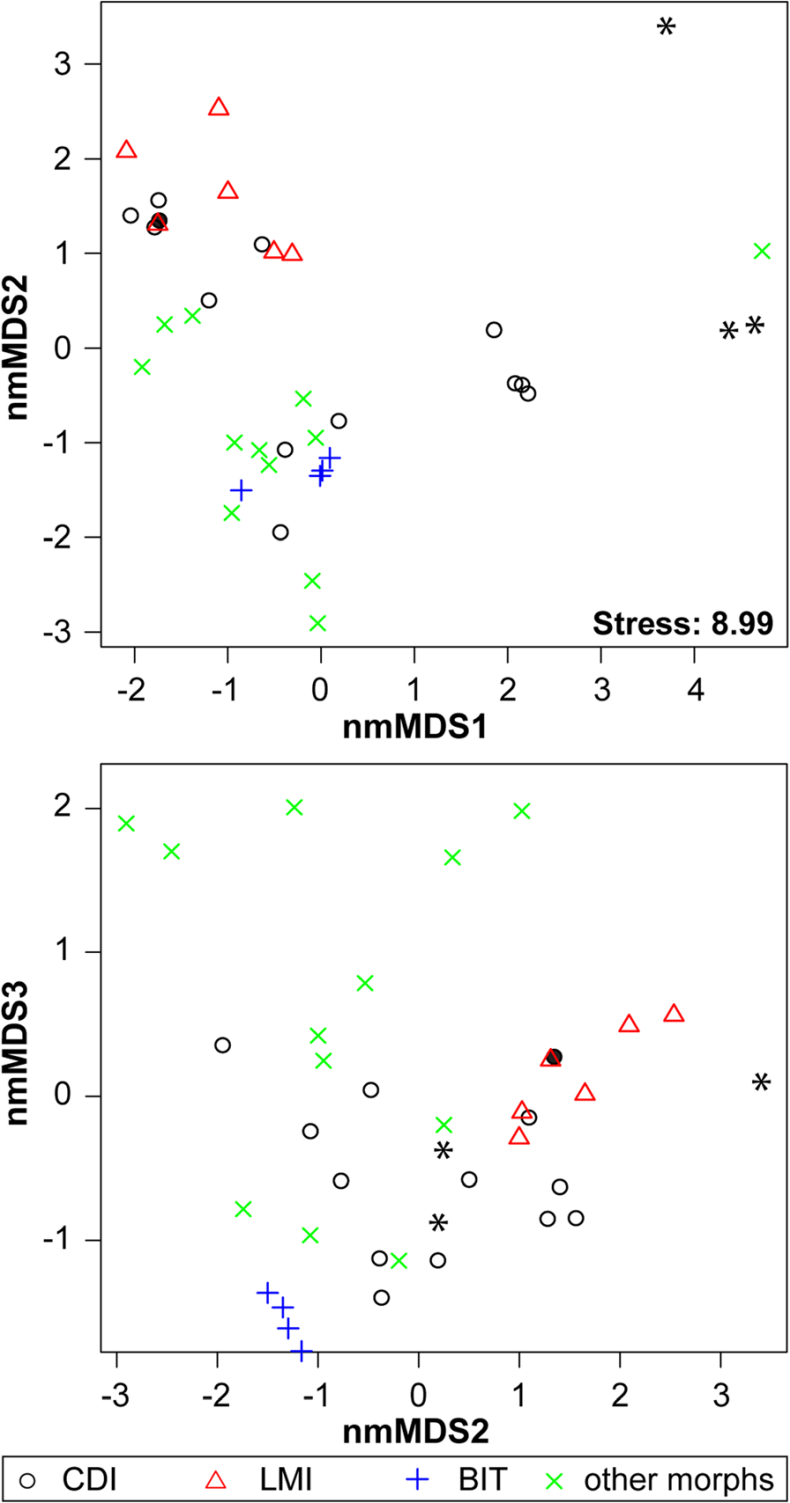
**Non-metric multidimensional scaling of morphologically-scored, African** ***Melanoides tuberculata*** **populations.** Morph codes are provided for invasive morphs, whereas native African morphs are lumped. Morphs CDI and LMI belong to clade 1, whereas all other material belongs to clade 2, illustrating that despite a deep phylogenetic split morphological overlap exists between both clades. Our study is the first to find morphological overlap between native and invasive *M. tuberculata* morphs, which hampers separating them based on shell morphology alone. The solid black circle indicates the position of lab-bred *F1* individuals of morph CDI; * indicates the position of organically coated CDI specimens that were not bleached before assessment.

Ordination of morphological characters of native and invasive *M. tuberculata* in morphospace resulted in limited stress (8.99), which suggests a reliable ordination result. This result confirmed the observations mentioned above (Figure 5) and that the invasive BIT morph of *M. tuberculata* displays limited morphological variability in comparison to CDI and LMI morphs. On a continental scale, an overlap exists in morphological variation displayed by native and invasive *M. tuberculata* morphs (Figure 5), although some of the overlapping populations belong to deeply divergent clades (Figure 2).

### Geographical modeling

**Figure 6:**
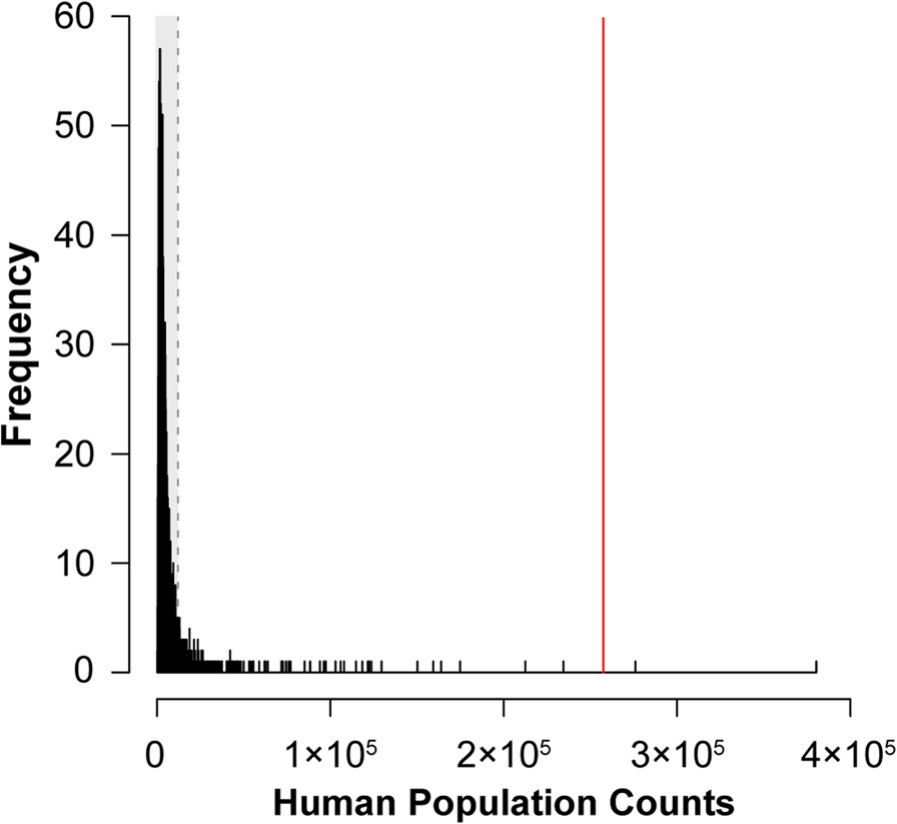
**Frequency distribution of summed human population counts in 6 randomly selected grid cells in sub-Saharan Africa.** The distribution was constructed from 10,000 sampling runs and compared to the observed summed human population counts in the 6 grid cells where invasions were observed (red line). Grey zone indicates the 95% samples with lowest human population counts. Only 2 of the 10,000 random sampling runs resulted in sums higher than the observed value, suggesting that invasions were non-random with respect to human population (as a proxy for anthropogenically-induced stress).

Invasive lineages of *Melanoides tuberculata* were documented from 6 major localities in 5 countries (Ede, Nigeria; Eseka, Cameroon; Kinshasa, Kisangani, DR Congo; Bujumbura, Burundi; Monkey Bay/Cape Maclear, Malawi). At >500 people/km^2^, all these localities are densely populated. To be conservative, we divided grid cells into a group with more, and one with less than 100 people/km^2^. Adjusted to the spatial scale of the NASA human population grid this resulted in 76,391 and 911,833 cells in the first and second groups, respectively. The probability that the 6 localities with invasions represent a random sample with respect to human population density is very small (*p* = 2.13e-07). Comparing the sum of human population counts from the grid cells where invasions were observed (~257,000 people) to the frequency distribution of summed human population counts from randomly selected grid cells in sub-Saharan Africa resulted in 2 out of 10,000 simulations having counts higher than observed (Figure 6). Modeling results indicate that colonization was non-random and mainly in highly populated, and hence anthropogenically disturbed areas.

## Discussion

### Reliability of phylogenetic data

Previous authors reported major incongruences between COI and 16S datasets, which prevented them from combining fragments into a concatenated analysis [22]. Inclusion of more specimens, sequencing of longer 16S fragments, and the use of more advanced software for alignment and phylogenetic inference (see methods) removed this incongruence (Figures 2 and 3). The branching pattern of major clades and the documentation of some lineages as invasive were consistently recovered.

### Phylogenetic patterns and biogeographic affinities

Phylogenetic analyses confirmed that *Melanoides* is paraphyletic and *M. tuberculata* polyphyletic [54], but interestingly, also the African endemic *Melanoides* species were revealed to be polyphyletic. Taxa endemic to Lakes Malawi and Mweru and the Lualaba River are part of clade 6, whereas others distributed more to the north in the Congo and Malagarasi Rivers belong to clade 4. As is the case for viviparids [57], the Congo system appears to play an important role in shaping biogeographical patterns for *Melanoides* species endemic to Africa, but additional sampling is required to evaluate similarities in the patterns of both taxa. Scrutinity is required to evaluate the position of *M. imitatrix* [54], and our phylogenetic reconstruction indicates a general need for more phylogenetic work (including nuclear markers) and taxonomic revisions of the genus *Melanoides*. Such revisions will ideally integrate molecular, conchological and anatomical analyses (see below).

Clades 1 and 2 of our phylogeny both contain Asian and African *Melanoides s*pecies, with different degrees of molecular divergence (Figure 2). Earlier authors [18,22,54] have suggested that the genus *Melanoides* probably has an African origin, and ancestral range reconstructions confirm the likelihood of this scenario.

The close and highly supported phylogenetic relationship of *M. admirabilis* from Tanzania and endemic *Melanoides* species from the Congo River (Figures 1 and 2), i.e. between gastropods from rivers flowing into and out of Lake Tanganyika, indicate the historic connection of biological provinces east and west of this lake. Close affinities of Malagarasi and Congo River mollusks have been suggested before, e.g. for *Potadomoides* [58], but we present the first molecular evidence for this hypothesis. Molecular evidence supporting close phylogenetic relationships also exists in Congo and Malagarasi fishes [59-61]. However, fish faunas of Lake Tanganyika appear to have seeded the fauna of the lake’s tributaries and outflow to a larger extent [60] than appears the case for mollusks [62,63]. The pattern of basin endemism and close genealogical relationships between taxa that occupy the same drainage basin also suggests that the dispersal capacities of the native, endemic *Melanoides* species are more restricted than those of the invasive *M. tuberculata* morphs. Moreover, the phylogenetic relationships between freshwater biota in the Malagarasi River and the Congo River and the independent *Melanoides* invasions to Lake Malawi and Lake Tanganyika provide testimony that the ecosystems of long-lived African lakes are permeable, at least periodically, to more widespread biota; see [62].

### Multiple invasions of *M. tuberculata* to Africa

Our phylogeny revealed that two deeply divergent clades of invasive, Asian *M. tuberculata* have contributed to the taxon’s continent-wide invasion of Africa (Figures 1 and 2). An invasive *M. tuberculata* morph (CDI) occurs in the Congo Basin, some 1,500 km away from the Malawi Basin, which is occupied by closely related invasive morphs (LMI_1-4_). These morphs are very closely related to two other morphs from Singapore (LSS, LSR) [21]. We cannot exclude that morph CDI invaded the Kisangani area directly via intercontinental transport from Asia, but given that the single CDI haplotype is shared with LMI populations successful dispersal of LMI specimens over hydrographic boundaries is more likely (which is also supported by ancestral range reconstructions). Other African representatives of this invasive clade (clade 5) are morphs CAE from Cameroon (Eseka), morph ZAK from the DRC (Kinshasa), and (not shown) morph ND from Nigeria (Ede) [20]. The molecular diversity in these African morphs suggests that multiple Asian representatives of clade 5 invaded Africa. Other morphs of clade 5 are invasive to America (morphs ARG from Argentina, TUM from Peru, BCI and USF from Florida, and *M. amabilis* from Martinique) and are genetically closely related to morphs with Asian origins, such as NAP from Singapore (Figure 2), morphs BAN and CHM from Thailand (Bangkok and Chiang-Mai, respectively; not shown), and morph ND from Indonesia (Lombok) [20,21]. These morphs (except for USF) are more distantly related to the CDI and LMI morphs, however. Another independent invasion occurred to Lake Tanganyika; this morph (BIT) is genetically nearly identical to morph USR from Singapore. Limited genetic diversity was observed within morphs CDI and BIT in comparison to that in invasive morphs from Lake Malawi, which suggests a single, potentially very recent, invasion for each of these morphs. Overall, our findings strongly indicate that multiple, independent invasions to Africa occurred, which may have resulted from increased aquarium trade [20,21]. Commercial activities with ornamental fish are reported for several of the localities where invasive *M. tuberculata* populations were recorded. More screening of other African *Melanoides* populations is required to fully document the geographic coverage of African invaders and the extent to which this coverage results from intercontinental introductions versus dispersal of invaders within Africa. Dispersal of LMI morphs to the north of Lake Malawi [22], to Lake Malombe [21], and further down the Shire River [24] suggests the morph will spread further through the Zambesi River system, and the occurrence of multiple CDI populations in various streams and ponds in the Kisangani area indicates that dispersal is ongoing there too.

### Morphological variation and ecophenotypy

Interestingly, the Asian invasive lineages resemble eachother somewhat in overall shell morphology, although they belong to deeply divergent clades (Figure 5, compare e.g. Figure 4A-C with 4M; Table 2). Moreover, our results document an overlap in the morphological variation of native and invasive *M. tuberculata*. This overlap does not exist for the invasive and native *M. tuberculata* from Lake Malawi [21], which may explain why these invasive populations were detected early on. Observations in the Malawi Basin may have raised the expectancy that both groups can be separated easily. However, this expectancy does not hold on a continental scale; morphs belonging to clades 1 and 2 of our phylogeny (Figure 2) cannot be readily distinguished from one another using shell characters. The existence of morphological overlaps underscores the need for integrated molecular-morphological approaches to assess cryptic *M. tuberculata* invasions. Difficulties to identify genetic lineages based on morphological observations in the field currently hamper efforts aimed at conservation and they have contributed to the uncertain taxonomy of African *Melanoides*.

Unfortunately, some of the characters that were used to distinguish LMI morphs from the native LMN morphs of Lake Malawi (the presence of a columellar band and the absence of axial ribs; [21]) cannot be used to diagnose the closely related CDI morph from the Congo Basin. Further examination of LMI specimens from Lake Malawi confirmed that LMI morphs have become more variable in these features than originally reported (Table 2; see also [21,24]). Moreover, some LMI populations display gigantism (Figure 4R,S), which is mainly observed in LMN morphs following parasitic castration [23]. However, the LMI specimens were not parasitized. A consistent morphological feature of CDI and LMI morphs is their very sharp apical angle, which is usually also observed in BIT specimens (Figure 4).

Although they have a genetic basis [18], shell features of the invasive *M. tuberculata* morphs are also subject to ecophenotypy. Differences in appearance relate partly to the organic layer that often covers shells, which may conceal differences between native and invasive *M. tuberculata* morphs (Table 2), and to actual shell morphology. The shell morphology of laboratory *F1* offspring of morph CDI resembles that of some LMI morphs from Lake Malawi more closely than the variable set of morphological features displayed by their parents (Table 2, Figure 5). This observation of marked phenotypic plasticity is somewhat surprising given the clonal reproduction, and that morphs of *M. tuberculata* are known to have colonized habitats halfway across the globe without morphological changes (e.g. morph PCD in Martinique and Japan; [17]). Morphological variation in wild-collected morph CDI, despite the lack of genetic diversity in the mitochondrial markers studied, is considerably larger than in LMI morphs (Figure 6), which hence confirms the great plasticity observed in experiments. More puzzling is that CDI individuals from the wild are usually coated with a thick organic layer that obscures most shell features. Perhaps morphological variation is correlated with other fitness-affecting traits, rather than having a direct selective advantage.

Our data are consistent with the suggestion that phenotypic plasticity and genetic variation resulting from serial introductions enhance the dispersal capabilities of invasive taxa [15]. However, various signals are observed: the LMI morphs that are spreading throughout the Malawi Basin display some genetic variability but seemingly limited plasticity whereas the opposite is observed for the CDI morph spreading in the Kisangani area (Figures 2 and 5). More research is required to elucidate this pattern and to look into the effects of plasticity and genetic variation on the success of invasion and dispersal. Although observed differences may relate to differences in ‘maturity’ of invasive populations and differences in the time of arrival to Africa, LMI morphs do not appear to have displayed elevated ecophenotyic plasticity before. Despite ecophenotypic variation, the apical angle appears to be a good diagnostic feature to select putatively invasive populations for molecular screening.

### Ecosystem deterioration and invasiveness

Eutrophication and pollution are known to increase the biomass of some opportunistic benthic mollusks including *M. tuberculata* [31,64]. *Melanoides* primarily inhabits sandy substrates [31], and it is perhaps not surprising that the invasive *M. tuberculata* strains were observed in areas of high anthropogenic disturbance. The Burundian shorelines of Lake Tanganyika near Bujumbura, where morph BIT was sampled, are very densely populated by humans and heavily disturbed by sedimentation and eutrophication [65,66] (Figure 2). Most of the cerithioids endemic to Lake Tanganyika, like many other taxa including fishes and ostracodes, are adapted to rocky substrates and increased sedimentation strongly affects their communities [67-69]. Simultaneously, it may open ecological opportunities for invasive taxa that prefer soft substrates. In Lake Malawi the invaders first occurred on the shallow sandy shores of Cape Maclear/Monkey Bay in the south, where increased eutrophication and sedimentation likewise occur [21,70]. The small streams and swamps bordering agricultural land near the city of Kisangani that are inhabited by morph CDI are likewise heavily polluted (Additional file 3: Table S3).

Geographical modeling indicated that the localities where invasive *Melanoides* populations were recorded represent a selective set of highly populated areas rather than a random sample of localities in sub-Saharan Africa with respect to human population (Figure 6). Our observations hence indicate that ecosystem deterioration and anthropogenic stressors increase the susceptibility of ecosystems to colonization by invasive species. However, several factors may contribute to this pattern. Anthropogenic ecosystem deterioration may create ecological opportunities for invaders, but larger human population densities and changes in human behavior also increase the frequency with which alien propagules arrive. Another aspect that potentially contributed to invaders being mainly observed in densely populated areas could be that these areas provide more accessible sampling spots (e.g. because of better infrastructure), and hence, chances to detect invasions may be greater in densely populated areas. Nevertheless, we sampled many localities outside urban areas as well (Table 1), and, hence, we consider it unlikely that the observed patterns would be caused solely by the last factor.

### Conservation concerns

Competitive interactions between invasive and native taxa may develop, certainly if the invader becomes established and migrates into previously unaffected environments. For example, in Lake Malawi negative spatial correlations are observed between the invasive LMI morphs and endemic *Melanoides* species, which suggests that competition may exist [24]. In the Kisangani area invasive *M. tuberculata* occurs in micro-sympatry with endemic cerithioids (*Potadoma*), and in Lake Tanganyika in close vicinity to habitats occupied by endemic cerithioids (*Tiphobia* and *Lavigeria*), but existing data do not allow assessing whether resource competition between endemic taxa and the invader occurs in these localities. Beyond regional endemism in *Potadoma* [30,71] endemic *Melanoides* species occur in the Kisangani area, but micro-sympatry was not observed between native and invasive *Melanoides* in this region.

Cerithioid gastropods are ecologically important and often represent a significant component of benthic communities in terms of diversity, biomass and nutrient recycling [69,72]. Lake Tanganyika, the Congo River and Lake Malawi are home to the most diverse freshwater cerithioid communities of the African continent. In Lake Tanganyika >10 endemic cerithioid genera occur and the latter two freshwater bodies are hot spots for endemic richness in native *Melanoides* species (12-13 [excluding taxa endemic to Lake Mweru] and 8 endemic species, respectively, accounting combined for two-thirds of the native *Melanoides* on the African continent; [30,31]). Recurrent invasions of non-indigenous species may result in competitive interactions between invaders and the native fauna, and beyond the elimination of native biodiversity, hybridization and genetic homogenization are eminent concerns for endemic biodiversity [2,20,73].

Multiple anthropogenically-induced environmental stressors regularly interact with each other, which results in a faster deterioration of ecosystem stability than anticipated from individual stressors alone [11]. The interactions between multiple stressors may directly or indirectly favor invasions and therewith biotic globalization [16]. The occurrence of some ecosystem stressors may hence increase the likelihood that additional stressors arise. In the benthic environments of long-lived lakes increased runoff, sedimentation and eutrophication are powerful agents of change [9,12,69] that may create new ecospace and ecological opportunities for invaders to settle. Intensified surveys with the aim of detecting biological invasions are required and perhaps the monitoring of aquatic invasions should, beyond biodiversity hotspots, also focus on stressed sites, where greater ecological opportunities for opportunistic, eurytopic invaders may be present. Better insights into the interactions between invasive and native taxa are urgently needed in long-lived lakes. Once invaders get established their effects are very difficult or nearly impossible to remove from ecosystems, so our best chance to restrict their impact perhaps lies in integrated ecosystem conservation and attempts to diminish the frequency of biotic introductions.

## Conclusions

Phylogenetic analyses of *Melanoides* specimens from sub-Saharan Africa with new methods removed previously existing inconsistencies between gene trees and documented the existence of multiple, independent invasions of Asian *M. tuberculata* to Africa. Our analyses furthermore indicated that *M. tuberculata* is polyphyletic, and the endemic, African *Melanoides* species as well, which highlights the need for more phylogenetic and systematic work. The areas that were invaded by Asian *M. tuberculata* all have high human population densities and anthropologically stressed aquatic ecosystems indicating that humans strongly determine colonization success. Affected ecosystems include long-lived Lakes Malawi and Tanganyika, and the Congo River, each of which has high endemic diversity in Cerithioidea. Morphological studies document overlaps in shell morphology between native and invasive *M. tuberculata* populations, and some invasive populations display great phenotypic plasticity, which appears to benefit their dispersal capabilities. The cryptic *M. tuberculata* invasions on a continental scale in Africa highlight the need for more concerted and integrated conservation strategies.

### Accession numbers

All DNA sequences used in this study are available from NCBI GenBank. Accession numbers are provided in Table 1.

## Additional files

Additional file 1: Table S1. Cycling conditions for polymerase chain reactions used to amplify gene fragments. Additional file 2: Table S2. Results of ancestral range reconstructions with Lagrange at nodes in the ingroup. Additional file 3: Table S3. Ecological data for localities where newly discovered invasive morphs of *Melanoides tuberculata* were found.
