# Supplementary material for: Recurrent camouflaged invasions and dispersal of an Asian freshwater gastropod in tropical Africa
Source: BMC Evol Biol. 2015 Mar 7;15:33. doi: 10.1186/s12862-015-0296-2 (PMC4373078; doi:10.1186/s12862-015-0296-2)
Supplement: Additional file 1: Table S1. — Cycling conditions for polymerase chain reactions used to amplify gene fragments. [file 12862_2015_296_MOESM1_ESM.docx]

**Additional file 1**

**Table S1. Cycling conditions for polymerase chain reactions used to amplify gene fragments.**

| **Step** | **Temperature (°C)** | **Time (min.)** | **Cycles** |
| --- | --- | --- | --- |
| **A) 16S and COI** |  |  |  |
| Initial denaturation | 95 | 1:00 | 1 |
| Denaturation | 95 | 0:30 | 35 |
| Annealing | 52 | 0:30 | 35 |
| Elongation | 72 | 0:30 | 35 |
| Final elongation | 72 | 3:00 | 1 |
| **B) COI (when protocol A was ineffective)** | | |  |
| Initial denaturation | 95 | 1:00 | 1 |
| Denaturation | 95 | 0:30 | 7 |
| Annealing | 52 (-1°C per cycle) | 1:00 | 7 |
| Elongation | 72 | 1:00 | 7 |
| Denaturation | 95 | 0:30 | 33 |
| Annealing | 42 | 1:00 | 33 |
| Elongation | 72 | 1:00 | 33 |
| Final elongation | 72 | 3:00 | 1 |
