# Supplementary material for: Recurrent camouflaged invasions and dispersal of an Asian freshwater gastropod in tropical Africa
Source: BMC Evol Biol. 2015 Mar 7;15:33. doi: 10.1186/s12862-015-0296-2 (PMC4373078; doi:10.1186/s12862-015-0296-2)
Supplement: Additional file 2: Table S2. — Results of ancestral range reconstructions with Lagrange at nodes in the ingroup. [file 12862_2015_296_MOESM2_ESM.docx]

**Additional file 2**

**Table S2. Results of ancestral range reconstructions with Lagrange at nodes in the ingroup.** These results were highly similar to those obtained with ape (Fig. 2). Relative probabilities of reconstructed scenarios are given for each node of interest in the format ‘upper branch|lower branch’. Within clade 3, nodes are represented from top to bottom as displayed in Fig. 2. Scenarios with a relative probability of <0.05 are not displayed, except if the scenario was included at another node with a likelihood of >0.05. Af = Africa; Am = America; As = Asia; O = Oceania. In summary, basal nodes typically have several scenarios with a somewhat similar likelihood; the relative probability is greatest for an African origin at the basal node; external evidence that American taxa within clade 5 represent historic Asian invasions (e.g. ref [20] in paper) results in the greatest relative probabilities for an Asian origin of this clade, and an Asian origin is also supported for the clade that comprises the BIT morph from Lake Tanganyika (Clade 3-2).

|  | **Af\|Af** | **Af\|Af+As** | **Af\|As** | **Af+As\|As** | **Af+As\|Af** | **As\|Af+As** | **As\|As** | **Af+Am\|Am** | **Am\|As+Am** | **Af\|Am** | **Af\|Af+Am** | **As\|As+Am** | **As\|Af** | **Af\|Af+O** | **Af+O\|Af** | **O\|O** | **O\|Af** | **O\|As** |
| --- | --- | --- | --- | --- | --- | --- | --- | --- | --- | --- | --- | --- | --- | --- | --- | --- | --- | --- |
| Basal node | 0.34 | 0.33 | 0.06 | 0.06 | 0.05 | 0.00 | <0.05 | 0.00 | 0.00 | 0.00 | <0.05 | 0.00 | 0.00 | <0.05 | 0.00 | 0.00 | 0.00 | 0.00 |
| Clade 1 | 0.29 | 0.17 | 0.00 | 0.06 | 0.06 | 0.20 | 0.10 | 0.00 | 0.00 | 0.00 | 0.00 | 0.00 | 0.00 | 0.00 | 0.00 | 0.00 | 0.00 | 0.00 |
| Clade 5 basal | 0.00 | 0.00 | 0.47 | 0.07 | 0.00 | 0.00 | <0.05 | 0.21 | 0.12 | 0.06 | 0.00 | 0.00 | 0.00 | 0.00 | 0.00 | 0.00 | 0.00 | 0.00 |
| Clade 5 upper | 0.44 | 0.00 | 0.00 | 0.00 | 0.11 | 0.00 | 0.00 | 0.00 | 0.00 | 0.00 | 0.42 | 0.00 | 0.00 | 0.00 | 0.00 | 0.00 | 0.00 | 0.00 |
| Clade 5 lower | 0.00 | 0.00 | 0.00 | 0.00 | 0.00 | 0.00 | 0.47 | 0.00 | 0.00 | 0.00 | 0.00 | 0.51 | 0.00 | 0.00 | 0.00 | 0.00 | 0.00 | 0.00 |
| Clade 6 basal | 0.00 | 0.00 | 0.00 | 0.00 | 0.00 | 0.00 | 0.00 | 0.00 | 0.00 | 0.00 | 0.00 | 0.00 | 1.00 | 0.00 | 0.00 | 0.00 | 0.00 | 0.00 |
| Clade 6 upper | 0.00 | 0.00 | 0.00 | 0.00 | 0.00 | 0.00 | 1.00 | 0.00 | 0.00 | 0.00 | 0.00 | 0.00 | 0.00 | 0.00 | 0.00 | 0.00 | 0.00 | 0.00 |
| Clade 6 lower | 1.00 | 0.00 | 0.00 | 0.00 | 0.00 | 0.00 | 0.00 | 0.00 | 0.00 | 0.00 | 0.00 | 0.00 | 0.00 | 0.00 | 0.00 | 0.00 | 0.00 | 0.00 |
| Clade 2 | 0.68 | 0.00 | 0.00 | 0.00 | 0.14 | 0.00 | 0.00 | 0.00 | 0.00 | 0.00 | 0.00 | 0.00 | 0.08 | 0.09 | 0.00 | 0.00 | 0.00 | 0.00 |
| Clade 3 base | 0.48 | 0.07 | 0.00 | 0.00 | 0.00 | 0.14 | 0.06 | 0.00 | 0.00 | 0.00 | 0.00 | 0.00 | 0.00 | 0.00 | 0.18 | 0.00 | <0.05 | 0.00 |
| Clade 3 - 1 | 0.00 | 0.00 | 0.00 | 0.00 | 0.00 | 0.00 | 0.00 | 0.00 | 0.00 | 0.00 | 0.00 | 0.00 | 0.00 | 0.00 | 0.00 | 1.00 | 0.00 | 0.00 |
| Clade 3 - 2 | 0.00 | 0.00 | 0.00 | 0.00 | 0.00 | 0.16 | 0.84 | 0.00 | 0.00 | 0.00 | 0.00 | 0.00 | 0.00 | 0.00 | 0.00 | 0.00 | 0.00 | 0.00 |
| Clade 3 - 3 | 0.00 | 0.00 | 0.00 | 0.00 | 0.00 | 0.00 | 0.00 | 0.00 | 0.00 | 0.00 | 0.00 | 0.00 | 0.00 | 0.00 | 0.00 | 0.00 | 0.75 | 0.25 |
| Clade 3 - 4 | 0.13 | 0.00 | 0.00 | 0.00 | 0.00 | 0.47 | 0.15 | 0.00 | 0.00 | 0.00 | 0.00 | 0.00 | 0.22 | 0.00 | 0.00 | 0.00 | 0.00 | 0.00 |
| Clade 3 - 5 | 0.36 | 0.00 | 0.00 | 0.00 | 0.62 | 0.00 | 0.00 | 0.00 | 0.00 | 0.00 | 0.00 | 0.00 | 0.00 | 0.00 | 0.00 | 0.00 | 0.00 | 0.00 |
| Clade 4 | 1.00 | 0.00 | 0.00 | 0.00 | 0.00 | 0.00 | 0.00 | 0.00 | 0.00 | 0.00 | 0.00 | 0.00 | 0.00 | 0.00 | 0.00 | 0.00 | 0.00 | 0.00 |
