# Supplementary material for: Recurrent camouflaged invasions and dispersal of an Asian freshwater gastropod in tropical Africa
Source: BMC Evol Biol. 2015 Mar 7;15:33. doi: 10.1186/s12862-015-0296-2 (PMC4373078; doi:10.1186/s12862-015-0296-2)
Supplement: Additional file 3: Table S3. — Ecological data for localities where newly discovered invasive morphs of Melanoides tuberculata were found. [file 12862_2015_296_MOESM3_ESM.docx]

**Additional file 3**

**Table S3. Ecological data for localities where newly discovered invasive morphs of *Melanoides tuberculata* were found.**

| **Specimen code** | **Locality** | **Latitude** | **Longitude** | **Habitat** | **Other mollusks** |
| --- | --- | --- | --- | --- | --- |
| CD01/1 | Makiso (DRC09-031) | N 0.51342 | E 25.20737 | small stream (± 1 m wide in dry season) separating agricultural land; substrate sandy, sides with reed, substantially polluted (high organic content, considerable runoff) | *Pila*, *Lanistes*, *Lymnaea* |
| CD05/1; CD06/1 | Makiso (DRC09-032) | N 0.51432 | E 25.20690 | small stream (± 1 m wide in dry season) separating agricultural land; substrate sandy, sides with reed, substantially polluted (high organic content), some trash within the water | *Pila*, *Bulinus* |
| CD07/1 | Kitenge (DRC09-033) | N 0.51724 | E 25.17857 | highly eutrophic agricultural pond with muddy to sandy substrate | *Lanistes* |
| CD09/1-2 | Ngengenge | N 0.62217 | E 25.28664 | turbid river (~10 m wide), substrate consisting primarily of sand and detritus | *Melanoides*, *Potadoma* |
| CD10/1-4 | Avokoko | N 0.46169 | E 25.34931 | small river (several meters wide); substrate: sand-gravel; mildly polluted | *Melanoides*, *Potadoma*, *Pila* |
| CD11/1-4 | Kpalala | N 0.46339 | E 25.36278 | small stream (few meters wide) with abundant macrophytes and a substrate consisting mainly of sand, some gravel and detritus | *Melanoides*, *Potadoma* |
| BI01/1 | Lake Tanganyika | S 3.37263 | E 29.33696 | nearby mouth of Ntahangwa river; shoreline with high deposition of sand/mud with a high content of organic material | *Coelatura*, *Pila*, *Pisidium* |
